# Numerical Model Driving Multi-Domain Information Transfer Method for Bearing Fault Diagnosis

**DOI:** 10.3390/s22249759

**Published:** 2022-12-13

**Authors:** Long Zhang, Hao Zhang, Qian Xiao, Lijuan Zhao, Yanqing Hu, Haoyang Liu, Yu Qiao

**Affiliations:** School of Mechatronics & Vehicle Engineering, East China Jiaotong University, Nanchang 330013, China

**Keywords:** rolling bearing, variable working conditions, dynamic analysis, WGAN-BP, cDNAP

## Abstract

Given the complexity of the application scenarios of rolling bearing and the severe scarcity of fault samples, a solution to the issue of fault diagnosis under varying working conditions along with the absence of fault samples is required. A numerical model-driven cross-domain fault diagnosis method targeting variable working conditions is proposed based on the cross-Domain Nuisance Attribute Projection (cDNAP). Firstly, the simulation datasets consisting of multiple fault types under variable working conditions are constructed to solve the problem of incomplete fault samples. Secondly, the simulation datasets are expanded by means of generating adversarial network to ensure sufficient samples for subsequent model training. Finally, cDNAP is used to obtain the cross-domain simulation projection matrix, which eliminates the variance in the distribution of measured and simulated sample features under varying working conditions. The experimental results of cross-domain for variable working conditions show that the diagnostic accuracy reaches up to 99%. Compared with DANN, DSAN, and DAAN domain adversarial neural networks, the proposed method performs better in bearing fault diagnosis.

## 1. Introduction

As a component of rotating machinery, rolling bearings play an essential role in the reliability and stability of the equipment. If bearing faults fail to be monitored in time, it is possible to cause casualties and massive property damage in severe cases [1,2]. Therefore, efficient monitoring and diagnostic are indispensable for the safe operation of mechanical equipment.

Numerous academics have conducted substantial research on bearing inspection methods and diagnostic techniques. For the bearing fault diagnosis methods under stationary working conditions, Huo et al. [3] proposed an adaptive multi-scale weighted permutation entropy for complexity analysis of time series, and the effectiveness of the proposed method for fault diagnosis under stationary working conditions was verified through experimental datasets involving different signal-to-noise ratios. Hou et al. [4] extracted fault features from the IMF components resulting from EEMD, followed by optimal feature se-lection through chi-square test and hierarchical clustering, and the method was validated on stationary working conditions. Chen et al. [5] proposed a deep residual network based on multi-task learning. Fault types and severity were considered to find the correlation in tasks. Fault types and severity under constant working conditions were successfully identified. As seen from the abovementioned works, it is evident that many achievements have been obtained with respect to bearing fault diagnosis under constant working conditions. However, there are few scenarios where the working conditions of actual mechanical equipment are constant, so investigating bearing fault diagnosis methods under varying working conditions is urgently necessary.

For fault diagnosis under variable working conditions, the methods available could be mainly categorified into three groups including signal demodulation [6,7,8], deep learning (DL) [9,10,11], and transfer learning (TL) [12,13,14].

In the case of varying speed, the rotational speed information can be retrieved using the key-phase device’s order tracking mechanism, and angular resampling is used to obtain stationary signals. However, the small installation space of the all-in-one device makes it difficult to install the components. A computational order tracking mechanism is suggested to alleviate the reliance on hardware. Choudhury et al. [15] proposed a tachometer-free order tracking technique based on a fast dynamic time-warping algorithm, which aligns the filtered signal with a constant frequency reference signal. It was possible to identify the bearing fault type under varying speed conditions. Order tracking technology independent of rotational speed information has severe harmonic overlapping as the speed fluctuates greatly. For this purpose, Hu et al. [16] proposed a generalized demodulation tacholess order tracking method for bearing fault diagnosis, using an improved cost function-based ridge extraction technology to extract rotational frequency harmonic components and reduce noise.

Generally speaking, the diagnostic results under varying working conditions depend significantly on the used signal analysis method. Intelligent fault diagnosis based on DL has been widely explored in recent years. As a data-driven method, DL models can automatically extract features with the help of massive historical data, with an inherent ad-vantage of gradually abandoning traditional signal processing in fault identification. Convolutional neural networks, deep auto-encoders, generative adversarial networks, recurrent neural networks, and domain adaptive networks are the prevailing models used for fault diagnosis in varied working conditions. Zhang et al. [17] proposed a new network of multi-mode CNN (MMCNN) to effectively extract rich and complementary fault features utilizing multiple parallel convolutional layers. In order to address the frequency-shift and amplitude-variation due to speed fluctuation, Han et al. [18] added batch normalization to each layer of deep neural networks acting as the basic framework for feature extraction. Softmax was then used to classify faults in the presence of speed fluctuation.

Despite the progress of deep learning-based methods in bearing fault diagnosis, they still suffer from some limitations. Traditional DL methods require sufficient labeled samples for model training. However, accessing adequate labeled data is costly, and labeled data are even unavailable for certain high-security devices. As such, traditional DL models exhibit poor diagnostic accuracy in the case of insufficient training data and thus model overfitting [19].

To solve the above problems, a GAN-based model was proposed to generate an adequate number of samples for bearing fault diagnosis [20,21,22,23]. After adversarial training of the GAN model, the trained generator yielded new samples with a similar distribution to the training samples. The results illustrate that a reliable diagnosis was made even with a few fault samples.

The GAN-based deep learning framework provides a solution to the fault diagnosis lacking enough training samples. However, the difference in feature distribution of various working conditions hinders the further improvement of fault diagnosis accuracy. TL is a new fault diagnosis method that maps data from two different kinds of distributions into a common space, minimizing the distance between different domains through KL scatter, maximum mean discrepancy (MMD), and Coral, thus improving the model’s generalization ability. Pei et al. [24] used WGAN-GP to generate source and target domain samples and input them into TL networks. Multi-kernel maximum mean discrepancies reduced the distance of edge distribution between source and target domains. The fault diagnosis under varying working conditions was thereby realized. In order to extract features from bearing vibration data under redundancy noise, Yang et al. [25] combined TL and DL to construct a deep residual shrinkage network by adding a soft threshold, where the joint maximum mean deviation (JMMD) and conditional domain adversarial (CDA) learning domain adapting network are utilized to align the source and target domains. Yu et al. [26] used transfer sparse coding (TSC) together with joint geometric and statistical alignment (JGSA) to extract features and align the difference between source and target domains, for the purpose of bearing fault diagnosis under varying working conditions. Wu et al. [27] denoised the collected vibration signal by EEMD. The denoised signal were then input to the DANN with an attention mechanism to extract domain-invariant features to achieve bearing fault diagnosis under varying working conditions. Yang et al. [28] developed multi-layer domain adaptive and pseudo-label learning regularization terms that reduced distribution differences and inter-class distances of features.

The use of TL offers fresh perspectives on bearing defect diagnostics under varying working conditions, but a significant problem lies in that acquiring source domain datasets with labeled information requires high experimental costs. Additionally, not all samples involving various fault types are available.

Numerical simulation has gradually developed into a potent tool for resolving engineering issues with the advance of computer performance. As a digital twin of the actual device, a dynamics model can simulate the dynamic behavior of complex devices. Thus, the dynamic model could be used to access simulated vibration signals of the bearings with different fault types under various operating situations. Therefore, the problem of insufficient data for model training in practical engineering applications is expected to be resolved. The potential advantages of dynamic simulation have been explored by several academics to diagnose bearing faults.

Yu et al. [29] proposed a simulation data-driven domain adaptive bearing fault diagnosis method, where the simulated source domain data come from the numerical simulation model of a rotor-bearing system. The diagnostic model gained from the source do-main was then used to determine the health status of bearings in actual equipment. Liu et al. [30] developed a bearing physics model to compensate for the difficulty of obtaining fault samples. Furthermore, a domain adversarial neural network model was proposed to achieve a high diagnosis accuracy for the scenario with small-sized samples. Ruan et al. [31] proposed a 5-degree-of-freedom bearing model to simulate the dynamic behavior of bearings with varied defect types and sizes, and the effectiveness of cross-domain fault diagnosis was confirmed by simulation datasets.

The scholars mentioned above took advantage of numerical simulation to realize bearing fault diagnosis with the help of simulated data. However, the above methods have a common drawback in the sense that they only consider the cross-domain from simulated to real data under the same working conditions. The cross-domain fault diagnosis from simulated to real data under variable working conditions has yet to be studied. Take into consideration the fact that the distribution difference between the simulated and real signals, in addition to the variable working conditions, makes it rather difficult for bearing fault diagnosis in real engineering. Few scholars have considered such two issues at the same time to arrive at a solution for practical bearing fault diagnosis under variable working conditions and limited sample scenarios.

Considering the practical problems of variable working conditions and lack of training samples, it is challenging to measure the distance between source and target domains by commonly used loss functions such as MMD. On the contrary, it is more applicable to execute cross-domain bearing fault diagnosis preceded with eliminating the redundant information due to variable working conditions. This paper proposes a cross-domain fault diagnosis method under varying working conditions with the aid of the nuisance attribute projection.

Nuisance attribute projection (NAP) is first proposed by Solomonoff [32]. The method was initially applied to speech and facial recognition to eliminate redundant information [33,34]. The NAP was introduced by Jiang et al. [35] to eliminate redundant information for bearing performance degradation assessment. The key of the NAP is constructing a projection matrix using the vibration signals of discrete load and speed to achieve the purpose of eliminating redundant information. To solve the problem of low accuracy of cross-domain fault diagnosis, NAP is introduced into TL to create the improved cross-domain nuisance attribute projection (cDNAP). The differences of feature distribution between the source and target domains under variable working conditions are eliminated by cDNAP.

In summary, the explicit dynamic model and cDNAP address the issues of limited samples and cross-domain differences under variable working conditions. The cross-domain bearing fault diagnosis method under varying working conditions is proposed. The main contributions of this paper include the following three sections:

1. A high-fidelity bearing dynamics model is constructed to simulate multiple working conditions and fault type samples. It provides a solution to the issue in which the failure samples are difficult to collect.

2. The cross-domain simulation projection matrix is obtained based on cDNAP. The projection matrix is used to eliminate redundant information between the simulated and measured signals under variable working conditions.

3. The proposed method can achieve high classification accuracy using a small amount of labeled real data, which can circumvent the practical constraints of data deficiency.

The remainder of this paper is arranged as follows: Section 2 briefly introduces the theoretical knowledge involved in the framework of the cDNAP model. The dynamic model and cDNAP are validated in Section 3. Two cases are analyzed in Section 4, the results show the outperformance of the cDNAP model, and three leading contrastive studies are performed to show the superiority of the cDNAP model. Finally, the conclusion is presented in Section 5.

## 2. Framework of cDNAP Model

This section presents the theoretical methods used in the paper, including the explicit dynamic method used for the dynamic model, the sample datasets expansion by WGAN, and the cDNAP that eliminates the differences between the source and target domains under variable working conditions.

### 2.1. Explicit Dynamic Method

The explicit dynamic based on nonlinear dynamic analysis must satisfy the conditions: displacement, velocity, and acceleration are known as t=0. The displacement, velocity, and acceleration of t+Δt are solved based on known quantities. The dynamic equation of the system of t can be estimated as [36]

(1) $$M{\ddot{x}}_{t}+C{\dot{x}}_{t}+Kx_{t}=F_{t}$$

where ${\ddot{x}}_{t}$, ${\dot{x}}_{t}$, $x_{t}$ represent the acceleration vector, velocity vector and displacement vector of the system nodes, respectively, M denotes the mass matrix of the system, C is the damping matrix of the system, K represents the stiffness matrix of the system, $F_{t}$ denotes the nodal load vector of the system.

$x_{t+\Delta t}$ of the expanding Taylor series at t can be modeled as

(2) $$x_{t+\Delta t}=x_{t}+\Delta t{\dot{x}}_{t}+\frac{\Delta t^{2}}{2}{\ddot{x}}_{t}+\ldots +\frac{\Delta t^{n}}{p!}x_{t}{}^{\left(p\right)}$$

where $x_{t}{}^{\left(p\right)}$ represents the p differential of $x_{t}$.

Combing Equation (2) with Equation (1), a quadratic differential equation is computed as follows

(3) $$x_{t+\Delta t}=x_{t}+\Delta t{\dot{x}}_{t}+\frac{\Delta t^{2}}{2}{\ddot{x}}_{t}$$

The derivation of Equation (3) can be modeled as

(4) $${\dot{x}}_{t+\Delta t}={\dot{x}}_{t}+\Delta t{\ddot{x}}_{t}$$

${\dot{x}}_{t}$ can be modeled as follow at [t−Δt,t]

(5) $${\dot{x}}_{t}=\frac{1}{\Delta t}(x_{t}-x_{t-\Delta t})$$

Similarly, ${\dot{x}}_{t-\Delta t}$ can be modeled as

(6) $${\dot{x}}_{t-\Delta t}=\frac{1}{\Delta t}(x_{t-\Delta t}-x_{t})$$

Simultaneous Equations (4)–(6). ${\ddot{x}}_{t}$ and $x_{t}$ can be modeled as

(7) $${\ddot{x}}_{t}=\frac{1}{\Delta t^{2}}(x_{t+\Delta t}-2x_{t}+x_{t-\Delta t})$$

${\dot{x}}_{t}$ and $x_{t}$ can be modeled as follow at [t−Δt,t+Δt]

(8) $${\dot{x}}_{t}=\frac{1}{2\Delta t}(x_{t+\Delta t}-x_{t-\Delta t})$$

Combining Equations (7) and (8), the iterative formula for each time point of the displacement values can be modeled as

(9) $$\left(\frac{M}{\Delta t^{2}}+\frac{C}{2\Delta t}\right)x_{t+\Delta t}=F_{t}-\left(K-\frac{2}{\Delta t^{2}}M\right)x_{t}-\left(\frac{M}{\Delta t^{2}}-\frac{C}{2\Delta t}\right)x_{t-\Delta t}$$

The stability condition for the explicit dynamic algorithm is

(10) $$\Delta t\leq \Delta t_{cr}=\frac{\tau_{n}}{\pi }$$

where $\tau_{n}$ represents the natural periods of vibration of the system. $\Delta t_{cr}$ is the critical value.

### 2.2. Generative Adversarial Net

GAN(Generative Adversarial Net) has been widely used as a mainstream data generation model. WGAN (Wasserstein Generative Adversarial Net, WGAN) [37] is used to address the problem of unstable generators by introducing the EM (Earth Mover) distance. EM can be modeled as

(11) $$W\left(P_{r},P_{g}\right)=\underset{\gamma \in \Pi \left(P_{r},P_{g}\right)}{\inf}E_{\left(x,y\right)\in \gamma }\left[\left\|x-y\right\|\right]$$

where x represents actual data, y represents generate data, (x,y) represents the sampling data from the γ, ‖x−y‖ represents the distance between x and y, $P_{r}$ and $P_{g}$ denote the data distribution, $\Pi \left(P_{r},P_{g}\right)$ denotes the set of joint distributions $\left(P_{r},P_{g}\right)$, γ represents a distribution.

Transforming Equation (11) into the form of a function through the KR (Kantorovich–Rubinstein)

(12) $$W\left(P_{r},P_{g}\right)=\underset{{\left\|f\right\|}_{L}\leq 1}{\sup}E_{x∼P_{r}}\left[f\left(x\right)\right]-E_{x∼P_{g}}\left[f\left(x\right)\right]$$

where ${\left\|f\right\|}_{L}\leq 1$ denotes the 1-Lipschitz function, *f* represents the distance mapping function.

The optimization objective of WGAN can be estimated as

(13) $$W\left(P_{r},P_{g}\right)=\underset{D\in \left(1-\mathrm{Lipschitz}\right)}{\max}\left\{E_{x∼P_{r}}\left[D\left(x\right)\right]-E_{x∼P_{g}}\left[D\left(x\right)\right]\right\}$$

where D∈(1−Lipschitz) represents the value of the discriminator within the range of 1-Lipschitz function.

The WGAN solves the issues of training difficulty, gradient dispersion, and model collapse by introducing the EM distance and 1-Lipschitz function. However, it brings the issue of gradient explosion, which directly affects the output performance of the discriminator. Gradient penalty (GP) is introduced into the WGAN [38,39]. The WGAN-GP enhance the performance of discriminator. GP can be modeled as

(14) $$\lambda E_{x∼P_{\hat{x}}}{\left[{\left\|\nabla_{x}D\left(x\right)\right\|}_{P}-K\right]}^{2}$$

where *K* denotes constant 1, $x_{r}$ follows the $P_{r}$ distribution, $x_{g}$ follows the $P_{g}$ distribution, $\hat{x}$ denotes random interpolation sample between $x_{r}$ and $x_{g}$. $\hat{x}$ can be modeled as

(15) $$\hat{x}=\varepsilon x_{r}+\left(1-\varepsilon \right)x_{g}$$

The generator and discriminator loss function of WGAN-GP can be modeled as

(16) $$\underset{D}{\min}V\left(D\right)=E_{x∼P_{g}}\left[D\left(x\right)\right]-E_{x∼P_{r}}[D\left(x\right)]+\lambda E_{x∼P_{\hat{x}}}{\left[{\left\|\nabla_{x}D\left(x\right)\right\|}_{P}-K\right]}^{2}$$

(17) $$\underset{G}{\min}V\left(G\right)=-E_{x∼P_{g}}\left[D\left(x\right)\right]$$

### 2.3. Cross-Domain Simulation Projection Matrix

**Figure 1:**
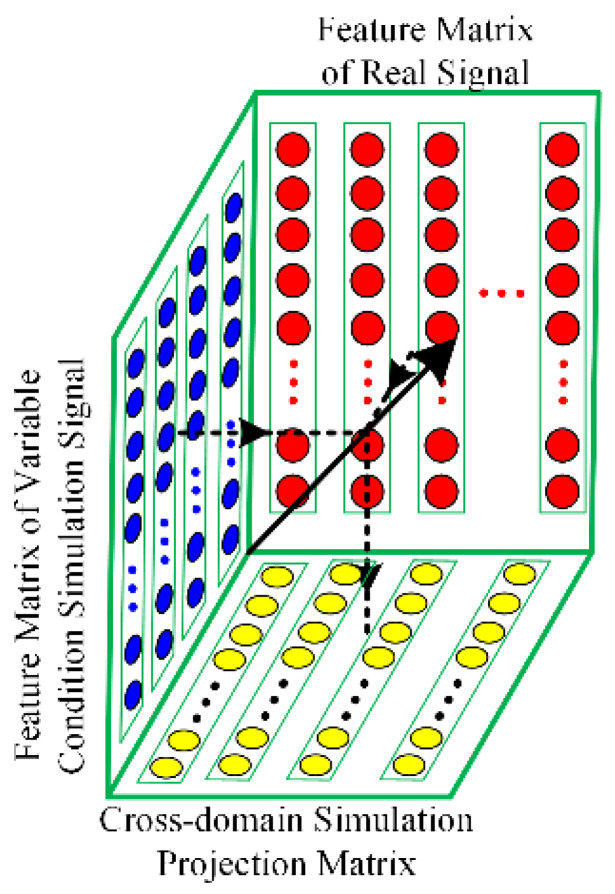
Principle of cross-domain nuisance attribute projection.

Due to the interference of variable working conditions and the difference between the source and target domains, the feature space of the raw signal contains fault and redundant information. The key of cDNAP is determining the simulation projection matrix, which eliminates the redundant information of variable working conditions and cross-domain. The theory of cDNAP is shown in Figure 1.

The N-dimensional feature space is defined. n represents the feature sample under different working conditions, the N×n feature matrix $F=[F_{1},F_{2},\ldots ,F_{n}]$ is constructed, feature matrix $F_{\mathrm{new}}$ after projection can be modeled as

(18) $$F_{\mathrm{new}}=P\times F$$

where P denotes projection matrix, P-dimensional is N×N, P can be modeled as

(19) $$P=I-\sum_{i=1}^{d}u_{i}u_{i}{}^{T}$$

where I represents the unit matrix, i denotes i nuisance attribute component, d represents the number of principal components, d smaller than the number of feature dimensions. The larger the d, the more redundant information is removed, and the fault information is likely to be eliminated. Therefore, the best d ensures that the most fault information and the least redundant information after projected.

The quality factor D denotes the sum of 2-norm distances of features between after and before projection. P is obtained by Equation (19), D is obtained by Equation (20), and the corresponding d is selected when the D is stable. D can be modeled as

(20) $$D={\sum_{ij}W_{ij}\left\|P(x_{i}-x_{j})\right\|}^{2}$$

where $W_{ij}$ represents the weight matrix for variable working conditions. When $x_{i}$, $x_{j}$ and $s_{i}\;$, $t_{j}$ are from the same working condition or the same domain, $W_{ij}$ is 0, and the opposite is 1.

The weight matrix W can be modeled as

(21) $$W_{ij}=\{\begin{array}{c} 1\\ 0\\ 1\\ 0 \end{array}\begin{array}{c} \mathrm{working}\;\mathrm{condition}\;x_{i}\neq \mathrm{working}\;\mathrm{condition}\;x_{j}\\ \mathrm{working}\;\mathrm{condition}\;x_{i}=\mathrm{working}\;\mathrm{condition}\;x_{j}\\ \mathrm{source}\;\mathrm{domain}\;s_{i}\;\neq \;\mathrm{target}\;\mathrm{domain}\;t_{j}\\ \mathrm{source}\;\mathrm{domain}\;s_{i}=\mathrm{target}\;\mathrm{domain}\;t_{j} \end{array}$$

where $x_{i}$, $x_{j}$ represent different working condition samples, $s_{i}$, $t_{j}$ represent source domain and target domain.

The solution of P in Equation (20) can be converted to obtain the leading eigenvectors of the following eigenproblem

(22) $$F(diag(W\times L)-W)F^{T}u_{i}=\lambda_{i}u_{i}(i=1,2,\ldots ,d)$$

where L represents the full 1-column vector of length *n*.

### 2.4. Flow Chart of Fault Diagnosis Method

**Figure 2:**
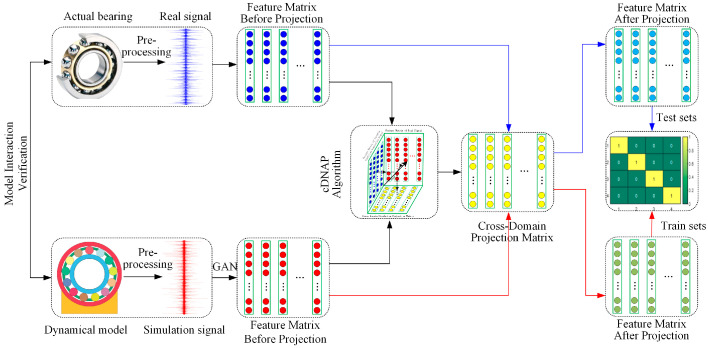
Flow chart of fault diagnosis based on cDNAP.

The proposed method achieves cross-domain bearing faults diagnosis under variable working conditions and insufficient samples. The flow chart is shown in Figure 2.

(1) A high-fidelity bearing dynamic model is constructed based on the actual situation, which simulates the vibration signals under multiple working conditions and fault types.

(2) The pre-processed simulated data are expanded by WGAN-GP. Based on the expanded simulation samples and a few measured samples, the cross-domain simulation projection matrix is obtained by cDNAP under varying working conditions. The features of measured samples are projected to eliminate the differences between the source and target domain under variable working conditions.

(3) The features of simulated sample are used as the training datasets, and the features of measured sample are used as the test datasets. They are finally achieving cross-domain bearing fault diagnosis under variable working conditions through extreme learning machine (ELM).

## 3. Validation of Bearing Dynamic Model and cDNAP

### 3.1. Establishment and Verification of Bearing Dynamic Model

(1) Establishment of the dynamic model

Each type of bearing has a unique character due to their material, size, lubrication, etc. [40]. This paper establishes a two-dimensional dynamic model of NU205 bearing. The fault is simplified into rectangular, and the signals of different working conditions are simulated. Bearing geometric parameters: pitch diameters 38.75 mm, rolling element diameter 6.75 mm, rolling element number 13, contact angle 0°. Material: outer ring, inner ring, and rolling element are GCr15. The cage is brass.

**Figure 3:**
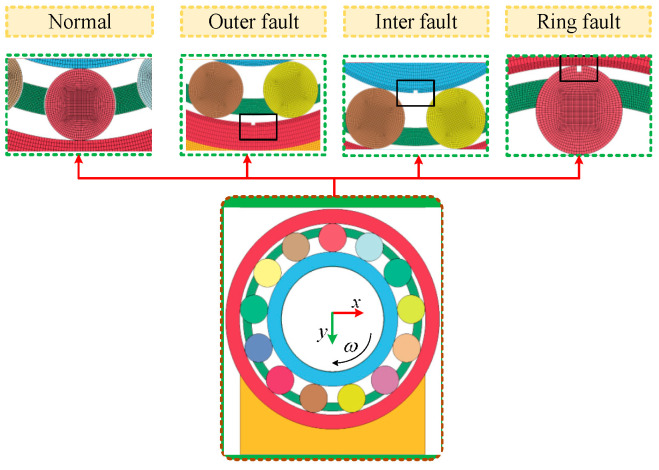
Bearing dynamic model.

Dynamic model boundary conditions: bearing housing with an interference fit outer ring, the radial load is applied on the inner ring inner surface, and the direction is Y positive. The rotational speed is used on the inner ring inner surface, and the direction is clockwise. The sampling frequency is 12 kHz. The mesh is refined to reflect better the vibration characteristics of the rolling element in contact with the inner and outer rings. The NU205 bearing dynamic model is shown in Figure 3.

(2) Verification of the bearing dynamic model

**Figure 4:**
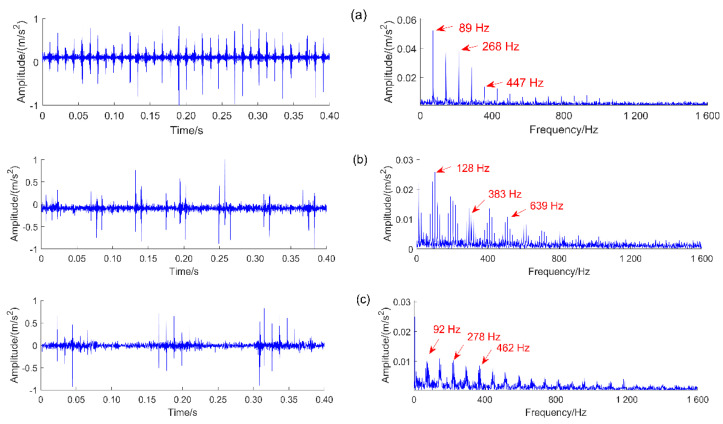
Simulation signals of different fault types and corresponding envelope spectra: (**a**) outer ring fault; (**b**) inner ring fault; (**c**) rolling element fault.

The reliability of the dynamic model ensures the correctitude of the study, so it is necessary to verify the validity of the dynamic model. The two-dimensional bearing model is point contact, so the amplitude of the vibration signals is large [41]. The vibration signals of normal, outer ring fault, inner ring fault, and rolling element fault are simulated when the speed is 1000 r/min. The outer ring fault, inner ring fault, and rolling element fault characteristic frequency are obtained by the envelope spectrum analysis, the results are shown in Figure 4.

The theoretical fault characteristic frequencies of outer ring fault, inner ring fault, and rolling element fault are obtained from Equations (23)–(25) [42]. They are 89.3 Hz, 127.2 Hz, and 92.8 Hz, respectively. The simulation signal fault characteristic frequencies of the bearing outer ring fault, inner ring fault, and rolling element fault are 89.0 Hz, 128.0 Hz, and 92.0 Hz, respectively. The relative errors are 0.34%, 0.63%, and 0.86%, respectively. The validity of the simulation model is proved.

(23) $$f_{o}=\frac{n}{2\times 60}(1-\frac{D}{d}\cos\alpha )Z$$

(24) $$f_{i}=\frac{n}{2\times 60}(1+\frac{D}{d}\cos\alpha )Z$$

(25) $$f_{b}=\frac{n}{2\times 60}\frac{d}{D}(1-{\left(\frac{D}{d}\right)}^{2}\cos^{2}\alpha )Z$$

where D denotes the rolling element diameter, n denotes the rotation speed, d is the pitch diameters, Z is the number of the rolling element, α is the contact angle.

### 3.2. Verification of WGAN-cDNAP

The obtained high-fidelity bearing dynamic model simulates the vibration signals of multiple loads, multiple speeds, and multiple fault types according to the actual requirements. The working condition types are shown in Table 1. N is the Normal state, OF is the outer ring fault, IF is the inter ring fault, and BF is the rolling element fault.

**Figure 5:**
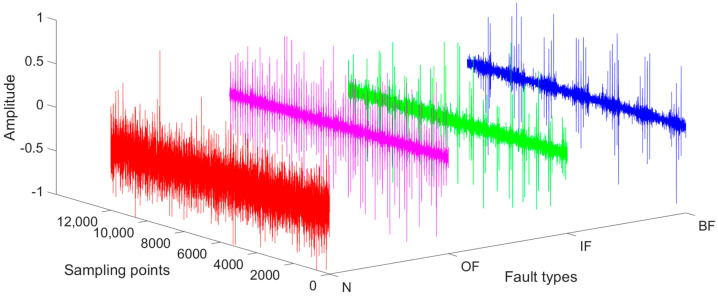
Simulation signals of normal, outer ring fault, inner ring fault, and rolling element fault.

There are discrepancies between the amplitude of the simulated and the measured signal due to the simplification of the dynamic model. The amplitude is mapped to [−1, 1] after the abnormal data of the simulated and measured signal are removed by the Pauta criterion. Simulated signals of four bearing health states are shown in Figure 5.

**Figure 6:**
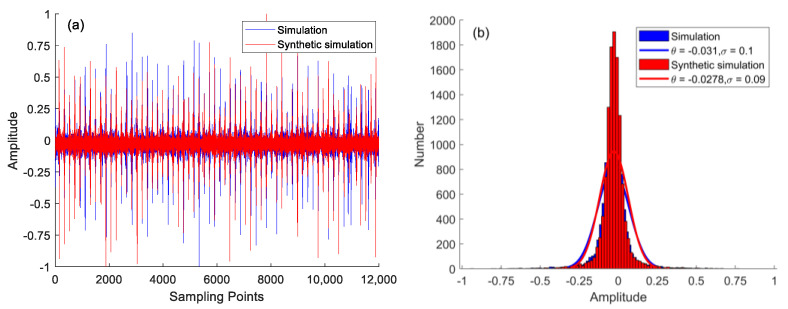
Signals before and after WGAN-GP: (**a**) simulation and synthetic simulation signals; (**b**) normal distribution and corresponding fit of simulation and synthetic simulation signals.

To obtain a sufficient number of samples, the simulation samples are expanded by WGAN-GP. The time-domain signals and fitted standard distribution histograms of the outer ring fault samples before and after expansion are shown in Figure 6. Figure 6a,b show the generated data has the same feature distribution as the raw signal.

The simulation and expanded datasets constitute the training datasets. To verify the effect of cDNAP to remove the redundant information of variable working conditions and the difference between the source and target domains, 29 features of the expanded samples are extracted. They are 11 time domain features, 8 wavelet packet decomposition node energies, 8 energy entropies when the wavelet level is three, amplitude spectrum entropy, and envelope spectrum entropy. The number of samples for each working condition is 50.

**Figure 7:**
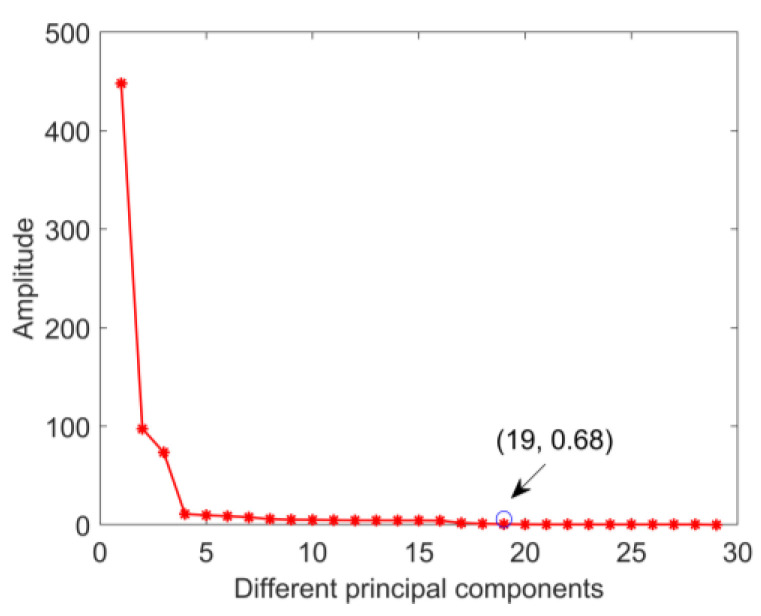
Amplitude of quality factor in different principal components.

**Figure 8:**
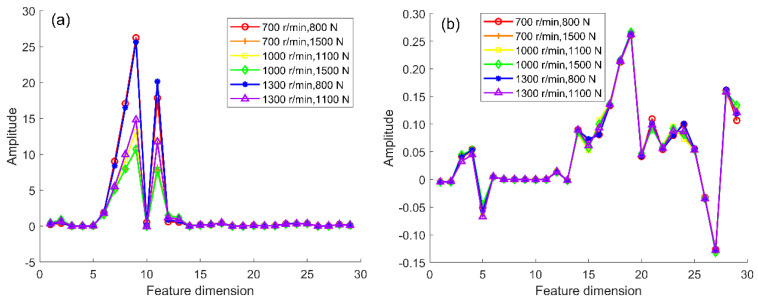
Distribution of features under variable working conditions before and after cDNAP: (**a**) before cDNAP; (**b**) after cDNAP.

The quality factor D determines the number of principal components and affects the consistency of data distribution after projection. The number of principal components when the quality factor tends to be stable can better eliminate the differentiation of feature distribution. The expanded simulation samples calculated by cDNAP to obtain the cross-domain projection matrices under different fault types: health state projection matrix (NDP), outer ring fault projection matrix (OFDP), inner ring fault projection matrix (IFDP), and rolling element fault projection matrix (BFDP). The projection matrix is multiplied by the sample features of the measured signal, which eliminates the feature distribution differences of the measured signal under the variable working conditions. The strength to eliminate the feature distribution difference between source and target domains under the variable working conditions is confirmed by the outer ring fault. The amplitude of quality factor in different principal components is shown in Figure 7, the result shows the amplitude of the quality factor decreases with the increase in the number of principal components and keeps stable when the number of principal components is higher than 19. Thus the number of principal components is determined to be 19. Furthermore, OFDP is obtained to eliminate redundant information. The results before and after the projection are shown in Figure 8.

In Figure 8a, the result shows that the distribution of features has some variability under different working conditions before the cDNAP projection, indicating that interference information from working conditions has an impact on fault diagnosis. Figure 8b shows that the feature amplitude of each sample is the same after cDNAP projection, indicating that the complex working condition information has been removed.

**Figure 9:**
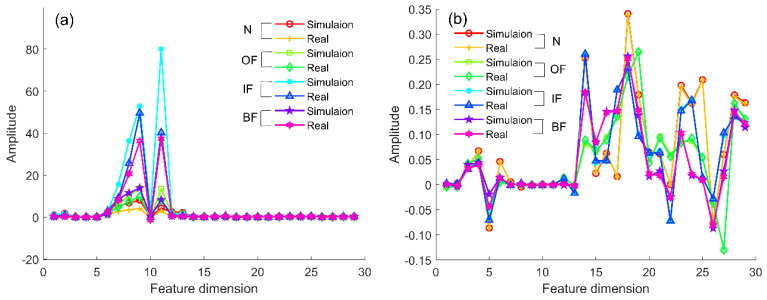
Distribution of features under four fault types before and after cDNAP: (**a**) before cDNAP; (**b**) after cDNAP.

Furthermore, to ensure that the differences between the source and target domains are eliminated—as well as the working condition information—the fault information is retained after projection, and the result for the four fault types is shown in Figure 9. In Figure 9a, the distribution of simulated and measured signal characteristics under the same fault type has some variations before cDNAP projection. The difference between the simulated and measured signals affects the accuracy of fault diagnosis. Figure 9b shows that the feature amplitude between simulated and measured is the same due to the differences are eliminated. More importantly, the distance among the different fault types is separated, which is an advantage for the bearing fault diagnosis.

## 4. Application Verification

**Figure 10:**
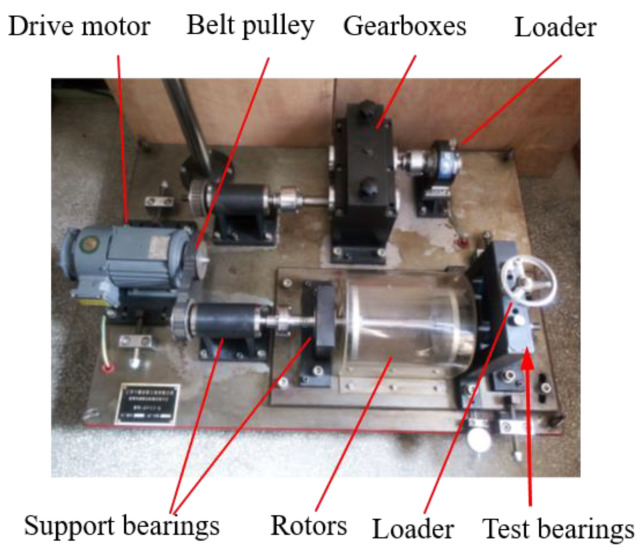
The test bench.

The test data come from the homemade rotating machinery simulation test bench shown in Figure 10. The test bench can simulate the vibration signal’s properties under bearing and gear failure, and this study makes advantage of its bearing failure simulation capability. To ensure that no external noise is generated during the experiment due to non-coaxial or friction, the rotating shaft and timing belt’s pulley are connected by coupling to compensate for an offset caused by incorrect installation and thermal expansion and ensure coaxial accuracy. The timing belt pulley makes the entire transmission process very smooth with minimal noise. The fastening bolts are adjusted to make the timing belt pulley run without noise before the experiment. The dowel-bearing is installed in the specified position to avoid abnormal vibration caused by the error of the assembly benchmark. The subsequent bearing failure simulation test will be conducted when the vibration, noise, and temperature rise are in normal conditions after a short operation of the assembled test bench.

The experimental bearing is a single row cylindrical roller bearing with detachable inner and outer rings, and the bearing model is N205EM. Bearing geometric parameters: outer ring diameter 52 mm, inner ring diameter 25 mm, pitch diameter 38.75 mm, rolling element diameter 6.75 mm, rolling element number 13, contact angle 0°, width 15mm, limiting speed 14,000 r/min, static load rating 28.7 kN, dynamic load rating 27 kN, the accuracy grade E. The outer ring is fixed during the experiment. The parameters of experimental bearing are consistent with the dynamic model, its sampling frequency is 12 kHz, and the sampling time is 10s. The bearing state of normal (N), outer ring fault (OF), inner ring fault (IF), and rolling element fault (BF) are processed. The condition types of bearings are shown in Table 1, which are consistent with the simulated conditions.

Four fault types are simulated by the dynamic model, and each fault type has nine different working conditions. Simulation time is 1 s, and the sampling points are 12,000. The datasets are expanded to 60,000 by WGAN-GP for each working condition. 1200 points as one cycle according to the speed and sampling frequency, total 50×1200 of samples, 29 features are calculated for each group of samples, getting 50×29 group features.

Given that the above datasets obey different distributions, a cross-domain fault diagnosis method under variable working conditions is proposed. Single condition and compound condition fault diagnosis strategies are used to reveal the effectiveness. The methods of different classifiers and feature extraction are used for comparison. Four different fault diagnosis strategies are support vector machine (SVM), ELM, ELM+NAP, and ELM+cDNAP. The main parameters of SVM: radial basis function, the value of loss function is 2, the value of kernel function is 1. The main parameters of ELM: the number of hidden neurons is 20, activation functions are sigmoid.

### 4.1. Bearing Fault Diagnosis of Single Working Condition

Working conditions include rotational speed and load. In the actual application of bearings, there is a common phenomenon that the speed or load is varies. Such as speed is constant and load is variation during the vehicle’s stable operation. The load is constant and the speed is variation during the car’s start/stop. Therefore, it is necessary for bearing fault diagnosis under single working condition. Based on the working condition types in Table 1, the bearing fault diagnosis under a single working condition are analyzed. The diagnosis results are shown in Table 2.

The training datasets (source domain) are simulated samples, and the test datasets (target domain) are measured samples. There is no overlap between the two datasets. The results of fault diagnosis are calculated by SVM and ELM, showing that the ELM method is better than SVM in fault diagnosis accuracy. However, the highest diagnostic accuracy is only 41.2%. The reason for the poor diagnosis accuracy is that the tremendous difference in feature distribution between simulated and measured signals under variable working conditions. The results show that the traditional method does not have engineered application value in cross-domain fault diagnosis under varying working conditions.

The fault diagnosis accuracy increased significantly after the NAP is introduced, and the average accuracy rate is 92.1%. The results show that the simulation projection matrix constructed by NAP reduces the difference in feature distribution between different working conditions. However, the results show differences between variable loads and speeds.

For variable load conditions, the average accuracy is 87.9% at a speed of 700 r/min, but the diagnostic accuracy drops to 85.2% when the target domain’s load amplitude is higher than the source domain. The average accuracy is 93.7% at a speed of 1000 r/min, but the diagnostic accuracy drops to 89.2% when the target domain’s load amplitude is between the source domains. The average accuracy is 95.2% at a speed of 1300 r/min, but the diagnostic accuracy drops to 94.5% when the target domain load amplitude is higher and lower than the source domain. It is clear from the above analysis that: (1) As the speed increases, the vortex motion becomes more stable, and the difference in vibration signal features becomes more minor under different loads; and (2) the fault diagnosis accuracy is affected by the intersection of the source and target domain datasets.

For variable speed conditions, the accuracy rate is higher under different loads, and the diagnostic accuracy is 95.6%, 92.5%, and 87.7% at speeds of 1300 r/min, 1000 r/min, and 700 r/min, respectively. The law of diagnostic accuracy under variable speed is the same as under variable loads, and both reflect the phenomenon of higher stability under high speed.

Considering the difference in feature distribution between the source and target domains under variable working conditions, the cross-domain simulation projection matrix obtained by cDNAP is multiplied by the simulated and measured signal, respectively, and the projected feature matrix is obtained. The simulated feature matrix is used as the training dataset, and the measured feature matrix is used as the test dataset for bearing fault diagnosis. The fault diagnostic accuracy under variable load and speed conditions reaches 99.3%. cDNAP eliminates the difference between source and target domains compared with NAP.

The method of cDNAP has higher diagnostic accuracy than NAP. However, the cDNAP relies on a small number of measured samples, and the NAP gets rid of the dependence on measured samples. Therefore, the appropriate feature processing method is selected according to the different application situations.

### 4.2. Bearing Fault Diagnosis of Compound Working Condition

In practical engineering applications, the speed and load change simultaneously are more general, such as the bearings in wind power gearboxes and shield tunneling machine, etc. Therefore, bearing status identification under compound working condition is more practical. The fault diagnosis under a compound working condition is studied based on the single condition fault diagnosis. The results are shown in Table 3.

The source domain is simulated samples, and the target domain is measured samples. The source domain consists of four working conditions, and the target domain working conditions differ from the source domain. The fault diagnostic accuracy of ELM is higher than that of SVM under compound working conditions. The results indicate that ELM learns more valid information from the training datasets. However, the accuracy of both diagnostics is not high, which is far from the demand for practical applications. The reason for the poor diagnostic accuracy is the excessive difference in feature distribution due to the dual influence of working conditions and cross-domain.

The fault diagnosis accuracy is greatly improved after the NAP is introduced. The average accuracy is 88.7%, which is reduced compared with the average accuracy under single working condition. The reason is the great difference between the source and target domains under variable working conditions. Further, the constructed simulation projection matrix eliminates redundant information about working conditions. However, the degree to which the differences are eliminated under different working conditions is varied. The fault diagnostic accuracy is 94.2% when the speed amplitude of target domain is larger than the source domain. The diagnostic accuracy is 88.8% when the speed amplitude of the target domain is within the source domain. The diagnostic accuracy is 82.9% when the speed amplitude of target domain is smaller than the source domain. The simulation projection matrix eliminates redundant information about working conditions while reducing the differences between the source and target domains. The diagnosis accuracy is low because the difference between measured and simulated samples is not considered. The method achieves fault type identification without access to the actual datasets.

The investigated cDNAP eliminates the distinctions between the source and target domains while retaining the information on the fault types. As a result, the diagnosis’ accuracy reaches 99%. Due to its dependence on the measured signals, the method has apparent diagnostic effects when applied to situations where fault samples can be obtained.

### 4.3. Comparative Experimental Analysis

**Figure 11:**
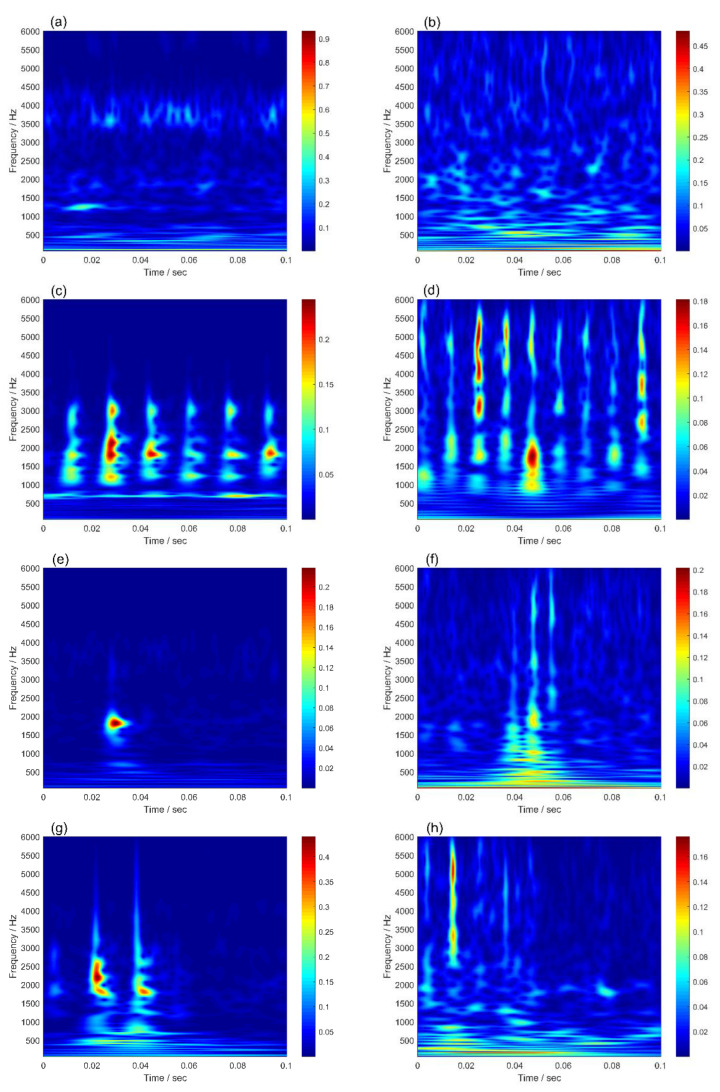
Time–frequency diagram of measured and simulated signals: (**a**) N of the measured signal; (**b**) N of the simulated signal; (**c**) OF of the measured signal; (**d**) OF of the simulated signal; (**e**) IF of the measured signal; (**f**) IF of the simulated signal; (**g**) BF of the measured signal; (**h**) BF of the simulated signal.

The research for variable working conditions and cross-domain fault diagnosis are favored by scholars—such as Domain Adversarial Neural Networks (DANN), Dynamic Adversarial Adaptation Networks (DAAN), and Deep Subdomain Adaptation Networks (DSAN)—which have achieved good results in the field of fault diagnosis under variable working conditions or cross-domain. However, the domain-adaptive method is only applied to variable working conditions or cross-domain, without successfully addressing the issue of concurrent change in both. The preceding strategies have limited practical application. The proposed method solves the problem of low accuracy of bearing fault diagnosis under variable working conditions and cross-domain from the perspective of eliminating redundant information. To illustrate the superiority of the proposed method over the mainstream domain adaptive methods, the diagnosis results of single and compound working condition are compared. Training datasets and test dataset samples of the DAAN, DANN, and DSAN are time–frequency maps obtained by wavelet analysis. The time–frequency diagrams of the simulated and measured signals under different fault types are shown in Figure 11. In Figure 11, the trend of the simulated and measured signals under each fault type is the same. There are differences between the simulation and the measured signal of the inner ring fault and rolling element fault. The reason is that the measured signal is affected by the transmission path.

The time–frequency graphs are input into the domain adaptive model for fault diagnosis. The ratio of training datasets to test datasets is 7:3. The training samples are 350, and the test sample is 150. Considering the diversity of loss functions, the loss function of DAAN is MMD, the loss function of DANN is LMMD, and the loss function of DSAN is Coral. The comparison results are shown in Table 4.

In Table 4, the deep domain adaptive neural network method does not have excellent cross-domain diagnosis results, and the diagnosis accuracy fluctuates wildly, which is unsuitable for application in engineering practice. The reason is that the loss functions of MMD, Coral, and LMMD are less effective in measuring the distance when the difference between the source and target domain distributions is significant under variable working conditions.

**Figure 12:**
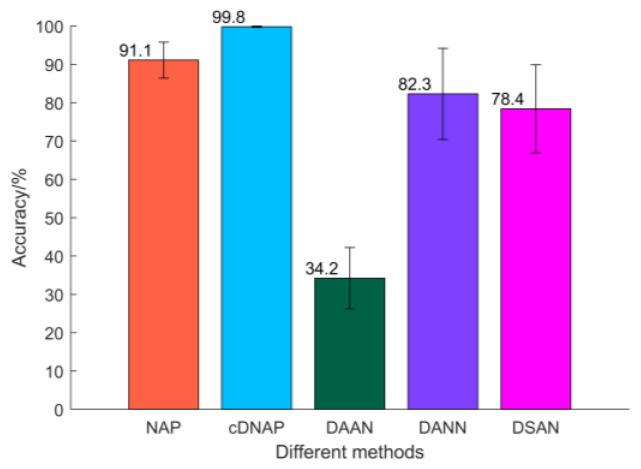
Average accuracy of different methods.

The average diagnostic accuracy of DAAN, DSAN, and DANN for MMD, Coral, and LMMD is visualized. The visualization results are shown in Figure 12. The results show that both NAP and cDNAP achieve better results in cross-domain fault diagnosis under variable working conditions, and the proposed diagnostic model is more stable.

## 5. Conclusions

A numerical model driving cross-domain fault diagnosis method under varying working conditions is proposed based on cDNAP. The issue of low fault diagnostic accuracy due to a shortage of fault samples is addressed.

(1) The dynamic model is constructed based on the bearing fault mechanism and dynamic performance. The vibration signals of multi-fault types under complex working conditions are simulated with the help of a high-fidelity dynamic model. It addresses the issue that fault samples are difficult to collect in practical engineering applications.

(2) The WGAN-GP is introduced to address the issue that the dynamic method requires a high computational cost to simulate a sufficient number of samples. WGAN-GP expands a large set of samples with consistent feature distribution to ensure generalization and robustness of the fault diagnosis model.

(3) The use of NAP solves the issue that the bearing fault diagnosis model relies on the measured samples and has superior engineering application value when the actual fault samples cannot be collected. The technique of cDNAP addresses the issue of inadequate generalization performance of diagnostic models with a few measured samples. The combination of dynamic simulation with NAP provides a new diagnostic idea for bearing faults. The matching method is selected according to the acquisition of actual fault samples to achieve the bearing fault diagnosis.

This study has achieved good fault diagnostic results in cross-domain fault diagnosis under variable operating conditions, but there are still some limitations: (1) Different bearing models need to be constructed with corresponding dynamic models due to the uniqueness of bearing types and operating conditions. (2) The features required for model training rely on manual extraction, and the merits of the features affect the fault diagnostic results. The following research will be conducted to address the limitations of the proposed method: (1) The construction method of an effective high-fidelity dynamic model will be studied. (2) The redundant attribute projection method will be combined with deep learning to achieve automatic feature extraction and then complete redundant information elimination and fault diagnosis to avoid the influence of human factors on fault diagnosis accuracy.

## Figures and Tables

**Table 1:** Working conditions of NU205.

Health States	Working Condition Types		
N; OF; IF; BF	800 N/700 rpm	800 N/1000 rpm	800 N/1300 rpm
	1100 N/700 rpm	1100 N/1000 rpm	1100 N/1300 rpm
	1500 N/700 rpm	1500 N/1000 rpm	1500 N/1300 rpm

**Table 2:** Fault diagnosis results of a single working condition.

Conditions	Source Domain		Target Domain		Accuracy/%			
	Speed/(Rad/s)	Load/N	Speed/(Rad/s)	Load/N	SVM	ELM	ELM+NAP	ELM+cDNAP
Variable load	700	800/1100	700	1500	25	33.2	85.2	100
	700	800/1500	700	1100	25	37.8	88.9	100
	700	1100/1500	700	800	24.5	36.2	89.7	99.9
	1000	800/1100	1000	1500	25	34.2	92.9	99.9
	1000	800/1500	1000	1100	25	39.4	89.2	100
	1000	1100/1500	1000	800	28	36.5	98.9	99.3
	1300	800/1100	1300	1500	25	35.1	94.5	99.9
	1300	800/1500	1300	1100	25	41.2	96.7	99.8
	1300	1100/1500	1300	800	25	38.8	94.5	99.6
Variable speed	700/1000	800	1300	800	25	33.4	97.7	99.3
	700/1300	800	1000	800	25	35.2	99.2	99.3
	1000/1300	800	700	800	25	36.2	91.8	99.9
	700/1000	1100	1300	1100	27	34.7	95.7	99.9
	700/1300	1100	1000	1100	20.5	35.4	84.9	100
	1000/1300	1100	700	1100	25	32.5	88.3	100
	700/1000	1500	1300	1500	24.5	36.2	93.5	99.9
	700/1300	1500	1000	1500	25	31.8	93.4	99.9
	1000/1300	1500	700	1500	25	32.8	83.6	99.9

**Table 3:** Fault diagnosis results of compound working condition.

Conditions	Source Domain		Target Domain		Accuracy/%			
	Speed/(rad/s)	Load/N	Speed/(rad/s)	Load/N	SVM	ELM	ELM+NAP	ELM+cDNAP
1	700/1000	800/1100	1300	1500	25	37.1	96.3	99.9
2	700/1000	800/1500	1300	1100	25	43.4	93.9	99.8
3	700/1000	1100/1500	1300	800	25	41.3	92.5	99.7
4	700/1300	800/1100	1000	1500	25	34.6	89.1	99.9
5	700/1300	800/1500	1000	1100	25	40.4	81.7	100
6	700/1300	1100/1500	1000	800	25	42.4	95.7	99.6
7	1000/1300	800/1100	700	1500	25	33.2	82.3	100
8	1000/1300	800/1500	700	1100	25	37.2	81.5	100
9	1000/1300	1100/1500	700	800	25	38.5	84.9	99.9

**Table 4:** Diagnostic results of comparison tests.

Conditions	Source Domain		Target Domain		Accuracy/%				
	Speed /(rad/s)	Load/N	Speed /(rad/s)	Load/N	ELM+NAP	ELM+cDNAP	DAAN	DANN	DSAN
1	700	800/1500	700	1100	88.9	100	42.2	77.3	67.8
2	1000	800/1500	1000	1100	89.2	100	37.0	69.7	70.5
3	1300	800/1500	1300	1100	96.7	99.8	31.5	98.8	92.3
4	700/1300	800	1000	800	99.2	99.3	29.2	75.0	67.8
5	700/1300	1100	1000	1100	84.9	100	39.0	75.0	77.8
6	700/1300	1500	1000	1500	93.4	99.9	25.0	90.7	90.2
7	700/1000	800/1500	1300	1100	93.9	99.8	28.7	99.0	94.2
8	700/1300	800/1100	1000	1500	89.1	99.9	50.0	90.0	83.7
9	1000/1300	1100/1500	700	800	84.9	99.9	25.0	64.8	61.3

## Data Availability

The data used to support the finding of this study are available from the corresponding author upon request.
